# Impaired expression of the COSMOC/*MOCOS* gene unit in ASD patient stem cells

**DOI:** 10.1038/s41380-020-0728-2

**Published:** 2020-04-23

**Authors:** Pauline Rontani, Olivier Perche, Louise Greetham, Nicolas Jullien, Bruno Gepner, François Féron, Emmanuel Nivet, Madeleine Erard-Garcia

**Affiliations:** 1 Aix Marseille University, CNRS, INP, UMR 7051 Marseille, France; 2 Orléans University, CNRS, INEM, UMR 7355 Orleans, France; 3 Department of Genetics, Regional Hospital, Orleans, France

**Keywords:** Autism spectrum disorders, Stem cells, Molecular biology, Autism spectrum disorders, Stem cells

## Abstract

Autism spectrum disorders (ASD) are complex neurodevelopmental disorders with a very large number of risk loci detected in the genome. However, at best, each of them explains rare cases, the majority being idiopathic. Genomic data on ASD derive mostly from post-mortem brain analyses or cell lines derived from blood or patient-specific induced pluripotent stem cells (iPSCS). Therefore, the transcriptional and regulatory architecture of the nervous system, particularly during early developmental periods, remains highly incomplete. To access the critical disturbances that may have occurred during pregnancy or early childhood, we recently isolated stem cells from the nasal cavity of anesthetized patients diagnosed for ASD and compared them to stem cells from gender-matched control individuals without neuropsychiatric disorders. This allowed us to discover *MOCOS*, a non-mutated molybdenum cofactor sulfurase-coding gene that was under-expressed in the stem cells of most ASD patients of our cohort, disturbing redox homeostasis and synaptogenesis. We now report that a divergent transcription upstream of *MOCOS* generates an antisense long noncoding RNA, to which we coined the name COSMOC. Surprisingly, COSMOC is strongly under-expressed in all ASD patients of our cohort with the exception of a patient affected by Asperger syndrome. Knockdown studies indicate that loss of COSMOC reduces *MOCOS* expression, destabilizes lipid and energy metabolisms of stem cells, but also affects neuronal maturation and splicing of synaptic genes. Impaired expression of the COSMOC/*MOCOS* bidirectional unit might shed new lights on the origins of ASD that could be of importance for future translational studies.

## Introduction

ASD are a heterogeneous group of neurodevelopmental disorders characterized by persistent deficits in social communication and interactions as well as restrictive, repetitive patterns of behaviors (DSM-5). So far, the precise mechanisms that underlie the complex pathophysiology of ASD remain obscure, which is largely due to the heterogeneous phenotypic presentation of the disease and the complexity of its inheritance. The genetic heterogeneity associated to ASD underscores the importance of identifying convergent pathways and molecular mechanisms that are responsible for these disorders. So far, collaborative studies between professionals/researchers and autistic adults together with shared resources suggest that over than 1000 genes are involved in ASD susceptibility [1–3]. However, at best, they explain less than 10% of the cases and the vast majority of ASD cases are idiopathic. The known functions of many of those candidate genes led the researchers to associate autism with an abnormal synaptic plasticity and so perturbations on neuronal/synaptic homeostasis [4, 5]. Although ASD cannot be reduced to neuronal and synaptic dysfunctions, it indicates that the identification of rare variants that could explain such disturbances during early development is important.

With the aim of identifying genes likely to contribute to the initial events leading to ASD etiology, we previously used olfactory stem cells (OSCs) isolated from nasal biopsies of ASD patients. These cells, displaying multipotent features [6, 7], are representative of early stages of ontogenesis and allow us to search for altered gene expression not observed in other cell types. Using pangenomic DNA microarray, we recently reported an under-expression of a transcript coding for the enzyme molybdenum cofactor sulfurase (MOCOS) in most ASD patients of our cohort [8]. MOCOS is mainly known for adding a sulphur on MOCO, a cofactor necessary for two enzymes - xanthine oxidoreductase (XOR) and aldehyde oxidase (AOX) - involved in purines metabolism [9]. We observed that reduced expression of *MOCOS* induces a hypersensitivity to oxidative stress, a perturbed synaptogenesis and an abnormal neurotransmission [8], features associated to neurodevelopmental deficits [4, 5, 10]. Given that MOCOS is involved in multiple biological and neurobiological functions, its loss of function is therefore coherent with phenotypic and aetiopathogenic heterogeneity in the ASD population.

In this study, we expand on that pioneer work and now report on the analysis of the upstream regulators of *MOCOS*. Recent advances in transcriptome sequencing have revealed long noncoding RNAs (lncRNAs) as a new class of genes able to regulate major biological processes, including synaptic development, maturation and plasticity [11–13]. However, understanding how lncRNAs control brain development and function remains elusive and no individual variant has been robustly associated to ASD so far [14–17]. With the help of frontline genetic tools, we have discovered that the promoter region of *MOCOS* can be read bidirectionally and induces the transcription of both *MOCOS* and an antisense lncRNA, which we named COSMOC. Antisense lncRNAs, usually poorly expressed and not conserved during evolution, were long considered as background transcripts. Nevertheless, we now know that they regulate almost all stages of gene expression, at the pre-transcriptional, transcriptional and post-transcriptional levels. LncRNAs can act in *trans*, on genes located on distant loci or other chromosomes, and in *cis*, on proximally positioned genes [18]. Therefore, COSMOC may be an interesting potential regulator of genes that could have critical roles in ASD.

To assess the possible implication of COSMOC on *MOCOS* expression and, more widely, its roles on cell metabolism, we used our initial model, namely human OSCs. We observed that (i) *COSMOC* transcription is necessary for the regulatory activity of this locus; and (ii) 10 out of 11 ASD patients display a drastic reduction of *COSMOC* expression, while an Asperger patient was only slightly perturbed. Then, we analyzed transcript variations when *COSMOC* was silenced and, further down, highlighted perturbations in lipid biosynthesis, chromatin organization, energy metabolism and neuron maturation, as well as insults aggravating the neurological outcome of ASD patients. We thus unveiled hidden functions of COSMOC that support the possible role that this lncRNA can play in ASD.

## Material and methods

### ASD patients and cell culture

Human nasal olfactory stem cells (OSC) from 11 patients and 11 age- and gender-matched control individuals without diagnosed neuropsychiatric disorders were taken under general anesthesia as previously described [8]. Primary OSCs and iPSC lines, as well as SH-SY5Y, U138-MG, HepG2, HEK-293 and Caco-2 cell lines were cultured as described in Supplementary Information.

### Derivation of neural progenitor cells and neurons from iPSCs

Neural progenitor cells (NPCs) were differentiated from human iPSCs (hiPSCs) by applying two different differentiation protocols as described in Supplementary Information. hiPSC-NPCs were differentiated into mature neurons with the BrainPhys^TM^ Neuronal Medium Kit (Stem Cell Technologies) following manufacturer’s instructions as specified in Supplementary Information.

### SH-SY5Y differentiation

SH-SY5Y differentiation was performed as described in Supplementary Information. For knockdown experiments, undifferentiated cells were incubated during 24 h with siRNA before the initiation of differentiation. The differentiation was stop at day 5.

### Generation of CRISPR-Cas9 mediated COSMOC-Knock-Out hiPSCs

CRISPR sgRNAs were designed using Crispor design tool (http://crispor.tefor.net) and cloned into cAB03 as described in Supplementary Information. The generation of COSMOC-Knock-Out hiPSC lines is detailed in supplementary Information,

### Transfection of cells and primers

Twenty-four hours after seeding, cells were transfected with Stealth RNAi™ siRNAs (Life Technologies) using standard protocols and primers listed in Supplementary Information and Supplementary Tables 1 and 2.

### Microarray gene expression analysis

OSC from two different healthy individuals were collected 48 h after treatment with siRNAs. Genome-wide transcriptional profiling was performed by Human Exon 1.0 ST arrays (Affymetrix) following manufacturer’s instructions. Each condition was performed in duplicate. Microarray data analysis is detailed in Supplementary Information. The microarray data (GSE122434) can be accessed in the Gene Expression Omnibus (GEO) repository at the National Center for Biotechnology Information.

### RNA isolation and real-time PCR analysis

RNA samples extracted from OSCs were tested with qRT-PCR as specified in Supplementary Information.

### Western blot analysis

Protein expression analyses in OSCs were performed using a standard protocol as described in Supplementary Information. Signals were visualized with ECL chemiluminescence kit (GE Healthcare) and quantified using ImageJ software.

### Oxidative stress induction, ROS assay and flow cytometry analysis

Twenty-four hours after seeding, cells were stressed for 2 or 4 h in the presence of H_2_O_2_ (500 µM) before RNA extraction. Intracellular ROS was measured by staining with CellRox® Deep Red Flow Cytometry Assay kit (Life Technologies) using FacsCanto cytometer (Becton Dickinson). Data were analyzed using FACS DIVA software.

### Immunocytochemistry

Cells were fixed in paraformaldehyde at room temperature and immunostainings were performed using standard procedures as described in Supplementary Information.

### Statistical evaluation

Statistical analyses were performed by using standard unpaired Student’s t-tests or one-way analysis of variance, and were carried out using Prism software (GraphPad Software, La Jolla, CA, USA) or Excel software (Microsoft, Redmond, WA, USA). All data are presented as mean ± s.d. and represent a minimum of three independent experiments with at least three technical replicates. Estimate of variance was performed within each group of data being statistically compared. A value of *P* < 0.05 was considered as statistically significant.

## Results

### COSMOC, an antisense long noncoding RNA upstream of *MOCOS*

**Fig. 1:**
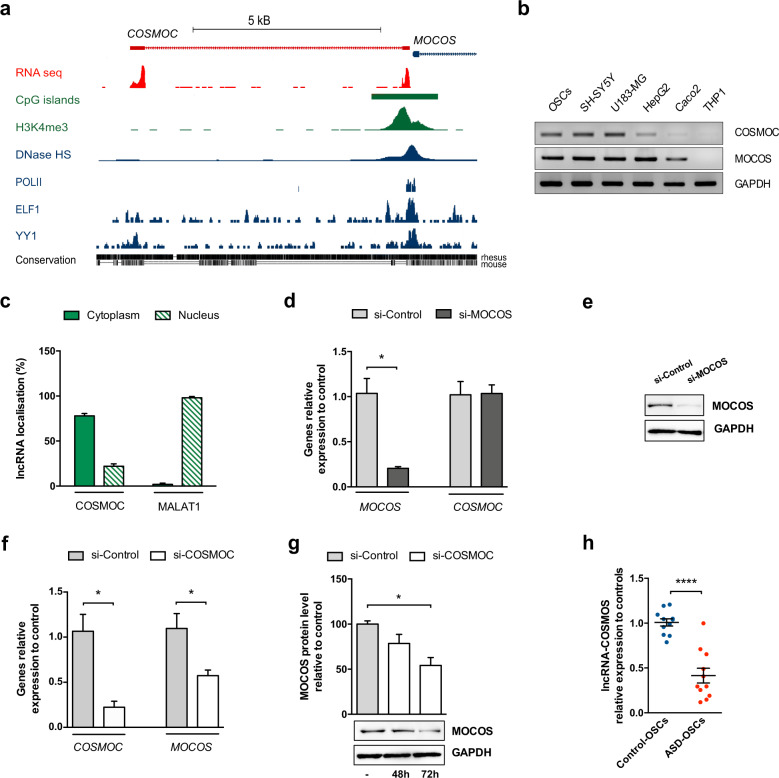
The lncRNA COSMOC regulates its neighbor gene *MOCOS* and its dysregulation affects all ASD patients of our cohort. **a** UCSC genome browser image depicting divergent transcription in the promoter region of the human *MOCOS* gene. The lncRNA COSMOC (also called RP11-49l11.1, transcript AC023043.1-201 or LOC101927809) spans 7454 bp and comprises 2 exons of 214 bp and 395 bp separated by a long intron of 6845 bp. It is transcribed in antisense direction to *MOCOS*. Tracks showing RNA-seq, CpG islands, Histone H3K4 trimethylation (H3K4me3) sites, DNase hypersensitivity spick clusters (DNase HS), RNA polymerase II binding (POLII) and binding sites for ELF1 and YY1transcription factors are from the human neuroblastoma cell line SK-N-SH. Base-level sequence comparison between COSMOC in human, monkey and mouse genomes indicates poor conservation in mice. **b** Agarose gel electrophoresis of *COSMOC* PCR products and Western blot analysis of MOCOS expression in a variety of cell lines. The specificity of the amplified products was confirmed by subsequent sequencing. GAPDH was used as a loading control for western blot. **c** Cell fractionation and qRT-PCR analysis of COSMOC expression in OSC. MALAT-1 is used as a control for nucleus/cytoplasm fractionation (*n* = 3). **d** qRT-PCR analysis of *COSMOC* and *MOCOS* expression in response to MOCOS knockdown in OSCs. Cells were transfected with scrambled interfering RNAs (si-Control) or interfering RNAs directed against MOCOS (si-MOCOS) (*n* = 4; Mann–Whitney test: *p < 0.05). **e** Western blot analysis of MOCOS expression in stem cells transfected with si-MOCOS. **f** qRT-PCR analysis of *COSMOC* and *MOCOS* expression in stem cells in response to COSMOC knockdown; (*n* = 5; Mann–Whitney test: *p < 0.05). **g** 72 h after treatment of OSCs with si-COSMOC, MOCOS downregulation was revealed by western blot. **h** qRT-PCR analysis of *COSMOC* gene expression in OSCs of ASD patients (ASD-OSCs) and matched healthy individuals (Control-OSCs), (*n* = 11; Mann–Whitney test: ****p < 0.0001). For all figures, *n* stands for number of biological replicates of OSC.

As a first attempt to apprehend the transcriptional control of *MOCOS* expression, we knocked down several putative upstream regulators, as detected using MatInspector (Genomatix) and TFBind software, but none of them successfully deregulated *MOCOS* expression (Supplementary Fig. 1a–c). We then used the data generated by advanced genomic tools, such as ENCODE and FANTOM5 and identified a divergent transcription in the *MOCOS* promoter region. This GC-rich region (GC- content: 65%) induces a transcription initiation on both sides with opposite orientations, generating an upstream antisense lncRNA near the 5’end of *MOCOS*, to which we coined the name COSMOC (Fig. 1a). COSMOC is a 609 nucleotides long and polyadenylated RNA (Supplementary Fig. 2a). Similarly to many other lncRNAs, it contains only two exons [19], separated by a long intron of 6845 bp and the noncoding anti-sense initiation of *COSMOC* is located 73 bp upstream of the transcription starting site of *MOCOS* (Fig. 1a and Supplementary Fig. 2b). COSMOC has no evidence of coding potential as indicated by its absence in protein database, a low PhyloCSF score [20] and no open reading frame longer than 150 nucleotides. Consistent with a crosstalk between coding and noncoding transcripts arising from the same locus [21], *COSMOC* and *MOCOS* exhibit a broad and similar pattern of expression across different human cell lines and tissues (Fig. 1b and Supplementary Fig. 2c). Of note, cell fractioning followed by qPCR analysis revealed that COSMOC can be found in the nucleus but it is predominantly located in the cytosol of OSCs (Fig. 1c), suggesting that its mode(s) of action on cellular mechanisms should not be reduced to a transcriptional regulator.

### COSMOC is under-expressed in ASD patient stem cells

To assess the functions of *COSMOC* and determine whether it can regulate *MOCOS* expression in a *cis* manner, we decided to use small interfering RNAs (siRNAs) to deplete both genes in human OSCs. When compared with cells transfected with scrambled small interfering RNAs (si-Control), siRNA directed against COSMOC (si-COSMOC) significantly diminished *MOCOS* transcript (Fig. 1d) and the corresponding protein by almost 50 % (Fig. 1e). In contrast, siRNA directed against *MOCOS* (si-MOCOS) did not affect *COSMOC* expression (Fig. 1f, g). Of note, this effect on MOCOS was found specific since COSMOC depletion did not alter the expression of *ELP2*, its upstream neighbor gene (Supplementary Fig. 2b, d).

Considering our previous observations that revealed the deregulation of MOCOS in OSCs established from our cohort of ASD patients (*8*) we then measured the expression level of *COSMOC* in these same cells. Strikingly, we observed significant lower levels of COSMOC expression in all patients, an Asperger patient being the exception, when compared with their respective age- and gender- match healthy controls (Fig. 1h). This data prompted us to study further COSMOC and identify how its downregulation could play a role in ASD pathology.

### COSMOC is involved in lipid metabolism, DNA regulation and redox homeostasis

**Fig. 2:**
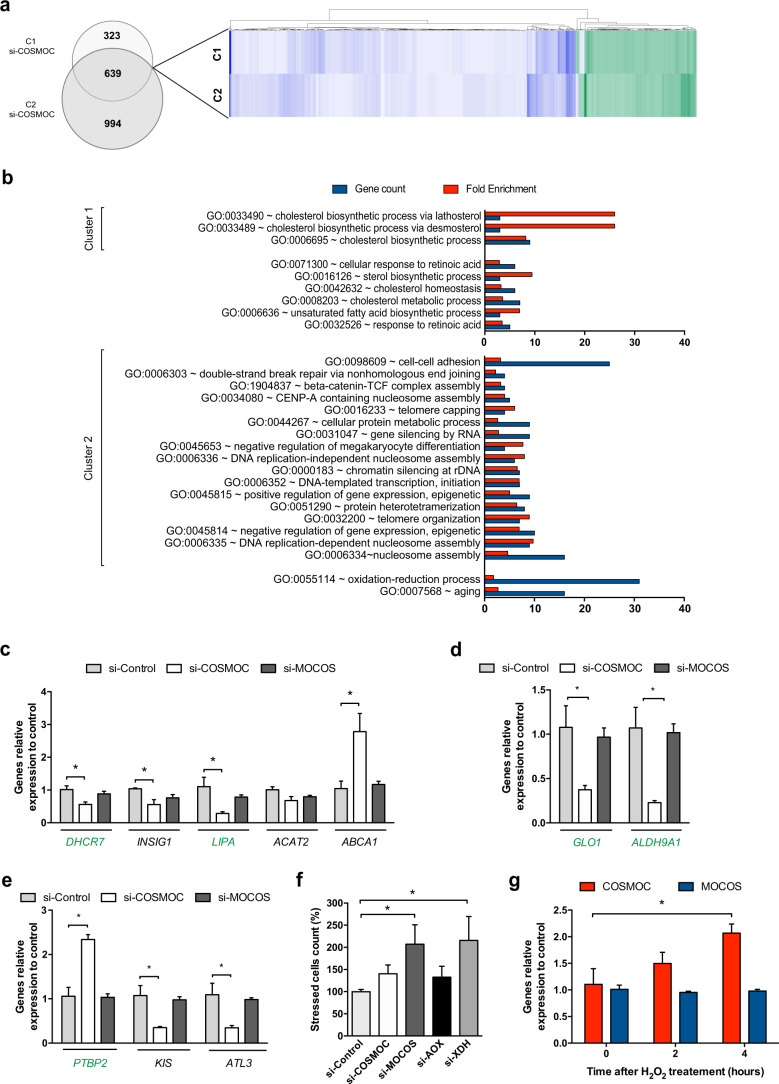
COSMOC knockdown alters lipid metabolism, DNA regulation and redox homeostasis. **a** Venn diagram summarizing the number of genes differentially expressed in OSCs of control subjects C1 et C2 transfected with si-COSMOC after normalization on control OSCs (*n* = 2). A two dimensional representation of the common dysregulated genes is visible in the heat map. **b** Gene ontology (GO) terms significantly enriched among differentially expressed genes in COSMOC-depleted cells. Red bars indicate the fold enrichment of the Gene Ontology (GO) and blue bars indicate the gene count. Two enriched clusters respectively enriched with genes involved in lipid and sterol metabolism or cholesterol biosynthesis and genes involved in nucleosome core, methylation and chromosome process were detected. The GO terms oxidation-reduction process and aging are not clustered or significantly enriched but represent a significant number of deregulated genes. **c**–**e** RT-qPCR validation of the transcriptome analysis. Dysregulation of a subset of genes involved in cholesterol biosynthesis process (DHCR7 INSIG1, LIPA, ACAT2 and ABCA1), redox homeostasis (GLO1 and ALDH9A1) and synaptic transmission or neural development (PTBP2, KIS and ATL3) was confirmed in OSCs deficient in COSMOC compared with cells treated with siRNA control. Highly similar results were obtained with both microarray and RT-qPCR techniques (*n* = 4; Mann–Whitney test: *p < 0.05). Genes known to be associated with ASD are written in green. **f** Quantitative analysis of oxidative stress in stem cells depleted in COSMOC, MOCOS, AOX, or XDH and comparison with cells treated with si-Control (*n* = 4; Mann–Whitney test: *p < 0.05). **g** RT-qPCR analysis of *COSMOC* and MOCOS expression after exposure of stem cells to H_2_O_2_ for 2 or 4 h (*n* = 4; Mann–Whitney test: *p < 0.05). For all figures, *n* stands for number of biological replicates of OSC.

To start identifying putative roles of *COSMOC*, we performed a transcriptomic study on OSCs from two healthy individuals in which we knocked down *COSMOC*. We found a pool of 639 mis-expressed genes, of which 150 were upregulated and 477 downregulated (Fig. 2a and GEO database GSE122434). As expected, *COSMOC*-depleted OSCs cultures showed that *MOCOS* was downregulated (fold change: −1.36 and −1.86 in individual 1 and 2, respectively). Of higher interest, among the top mis-expressed genes upon COSMOC depletion, eleven of them - *ALDH9A1, DHCR7, ENPP2, FABP3, GLO1, GLUL, LIPA, MOCOS, PPT1, PTBP2, SEMA5A* - have been previously associated with ASD (*1-4*; https://gene.sfari.org/). Furthermore, DAVID-based gene clustering analysis revealed two significantly distinct groups (Fig. 2b). The first cluster (enrichment score 3.21) included genes involved in lipid and sterol metabolism or cholesterol biosynthesis, whereas the second one (enrichment score 2.94) was composed of genes associated to nucleosome core, methylation and chromosome processes. Under the designation “Oxidation-reduction process”, gene ontology classification indicated an altered detoxification machinery (Fig. 2b and Supplementary Table 3). Dysregulation of several ASD-associated genes and some genes involved in synaptic transmission or neural development were further validated by qPCR analyses (Fig. 2c–e). These data suggested that COSMOC modulates the functioning of relevant cellular mechanisms formerly implicated in ASD. Of note, MOCOS downregulation did not alter the panel of genes we tested, indicating that putative functional effects of COSMOC downregulation in ASD may act through MOCOS-independent mechanisms.

In light of our previous data showing an increased ROS production in ASD patient-derived OSCs, we next decided to measure the number of stressed cells in *COSMOC*-silenced conditions. We observed a mean increase of 39.7% (Fig. 2f and Supplementary Fig. 3a–c) in the number of stressed cells from those cultures treated with si-COSMOC. Since depletion in MOCOS and MOCOS-dependent enzymes (i.e., AOX and XDH) induces ROS (Supplementary Fig. 3a), we next treated OSCs for various time durations with hydrogen peroxide (H_2_O_2_), a potent catalyst of ROS. By doing so, we assessed whether the disturbed redox homeostasis was a consequence of *COSMOC* depletion or *COSMOC*-induced MOCOS exhaustion. As shown in Fig. 2g, cells started transcribing *COSMOC* before *MOCOS*, suggesting that, within the *COSMOC/MOCOS* duo, *COSMOC* is the first to respond to oxidative stress.

### Modulated expression of COSMOC during neural differentiation

**Fig. 3:**
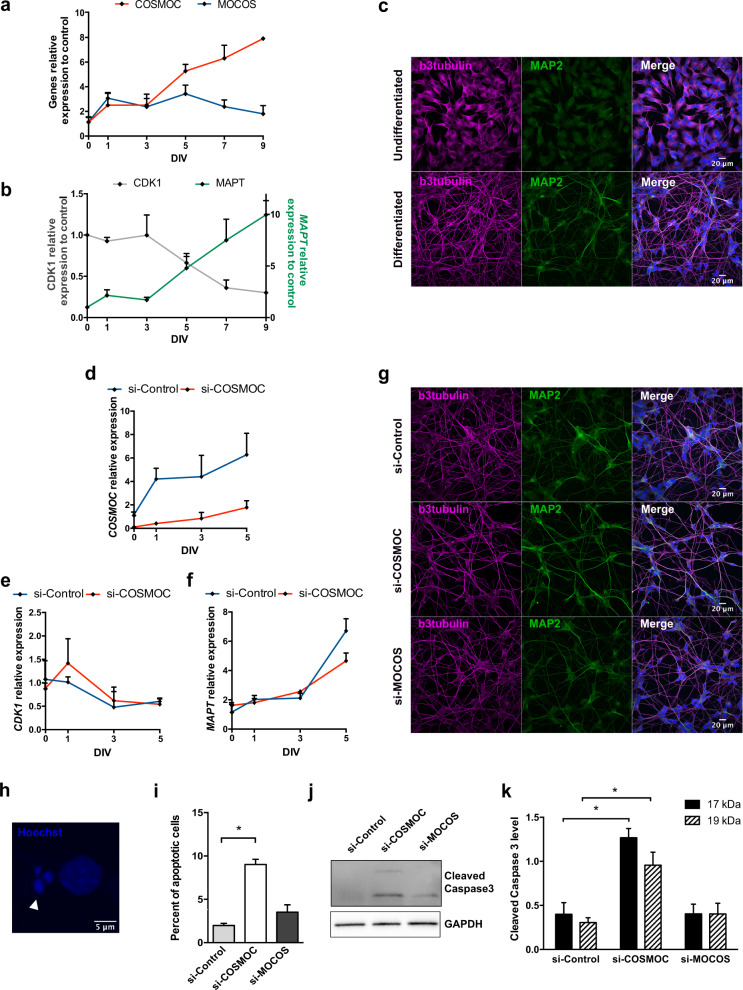
COSMOC is required for neuronal survival. **a** RT**-**qPCR analysis of COSMOC and *MOCOS* expression during neuronal differentiation of SH-SY5Y cells for 9 days in vitro (DIV). Gene expression was normalized to time-point 0 (*n* = 3). **b** Neuronal differentiation was confirmed by RT-qPCR analysis showing decreased expression of the cell division marker *CDK1* and increased expression of the neuronal marker *MAPT*. Data were normalized to time-point 0 (*n* = 3). **c** Immunocytochemistry with the neuronal differentiation markers βIII tubulin and MAP2 on undifferentiatied and differentiated SH-SY5Y cells at 9 days. **d** RT-qPCR analysis showing effective depletion of COSMOC in SH-SY5Y cells during the first 5 days of neuronal differentiation. SH-SY5Y cells treated with si-COSMOC were compared with cells incubated with scrambled siRNA and data were normalized to time-point 0 (*n* = 3). **e,** **f** RT-qPCR confirmation of effective neuronal differentiation of SH-SY5Y depleted in COSMOC expression during the first 5 DIV. The expected inverse correlation between *CDK1* expression (**e**) and *MAPT* expression (**f**) was observed in SH-SY5Y treated with si-COSMOC and those treated with scrambled siRNA. Data were normalized to time-point 0 (*n* = 3). **g** Immunocytochemistry showing βIII tubulin and MAP2 staining of SH-SY5Y cells treated with siCOSMOC, siMOCOS or control siRNA at 5 DIV. **h** Analysis of cell death by nuclear staining with Hoechst. Example of a nucleus counted as apoptotic after staining with Hoechst (leftmost) and compared with two adjacent normal nuclei. **i** Quantification of apoptotic nuclei at 5 DIV in SH-SY5Y knockdown for COSMOC, MOCOS or control siRNA (*n* = 4; Mann–Whitney test: *p < 0.05). Representative Western blot (**j**) and quantification (**k**) of cleaved products of caspase-3 (17 and 19 KDa) at 5 DIV in SH-SY5Y cells depleted in COSMOC, MOCOS or control siRNA. GAPDH was used as control. (*n* = 4; Mann–Whitney test: *p < 0.05). Data are represented as mean ± SEM. For all figures, n stands for number of biological replicates of SH-SY5Y cells.

LncRNAs being known as key regulators of neural differentiation [11, 22], and ASD being primarily associated with neurodevelopmental disorders, we next examined whether *COSMOC* modulation could impact on neurodevelopmental-like processes by using in vitro strategies. First, and to test its potential role during neuronal differentiation/maturation we analyzed the dynamic expression of *COSMOC* in SH-SY5Y, a human neuroblastoma cell line able to differentiate into a homogeneous population of neuron-like cells [23]. As shown in Fig. 3a–c, SH-SY5Y cells exhibited a rapid and progressive increase of *COSMOC* expression during the differentiation procedure, reaching an 8-fold increase in 9-day old differentiated neuron-like cells. In parallel, MOCOS expression did not change throughout the differentiation. Of importance, and validating the acquisition of a post-mitotic phenotype in those differentiated cells, we observed an inversed correlation between *CDK1* expression and *MAPT* expression (Fig. 3b), with *CDK1* being involved in cell cycle whereas MAPT is critical for neuronal maturation. Neuronal differentiation was further confirmed by the acquisition of morphological changes resembling neuronal-like morphology but also by the expression of the neuronal marker MAP2 as detected by immunocytochemical analysis (Fig. 3c).

Considering our initial observations we next investigated the effects that *COSMOC* knockdown may have on SH-SY5Y-based neuronal differentiation (Fig. 3d and Supplementary Fig. S4a–g). During the first 5 days of differentiation, no change was observed in *COSMOC*-downregulated differentiating neurons when compared with scrambled-siRNA treated ones (Fig. 3e–g). However, from day 6 of differentiation and onward, *COSMOC* knockdown caused morphological changes including cell shrinkage. Because these changes are important features of programmed cell death in neurons, we scored alterations in nuclear morphology and caspase3 activation (Fig. 3h–k). At Day 5, we observed a significant higher number of apoptotic nuclei in *COSMOC*-downregulated neuronal-like cells, with a condensed and fragmented chromatin (Fig. 3h, i) while cell death was confirmed by a substantial increase in active caspase-3 (Fig. 3j, k). Of note, despite a slight increase in the number of apoptotic bodies, no significant impact on neuronal cell death was observed upon *MOCOS* downregulation (Fig. 3i), suggesting that COSMOC may act on neuronal cells in a *MOCOS*-independent manner. In that regard, it is worth noting that, as specified in our previous report, all-except-one ASD patients of our cohort are suffering from a loss of brain volume, atrophy or dysmorphy [8]. These results indicate that COSMOC could be a critical player in processes controlling neuronal survival and may participate to neurodevelopmental processes.

Therefore, in view of the fact that ASD pathogeny is perceived as a neurodevelopmental disorder that takes place during the initial steps of brain development, we asked whether COSMOC might be able to interfere with early developmental processes. Using the human induced pluripotent stem cells (hiPSC) technology, we first investigated the dynamic expression pattern of *COSMOC* during differentiation into neural progenitor cells (NPC). Interestingly, using two distinct induction protocols, we observed a burst of *COSMOC* expression as early as day 1 after neural induction of hiPSCs. This moderate increase was followed by a rapid but progressive decline during the next few days of differentiation until *COSMOC* expression became undetectable in differentiated NPCs (Supplementary Fig. 5a, b). The efficacy of our differentiation strategies was confirmed by PAX6, SOX2 and NESTIN immunostaining in hiPSC- derived NPCs and TUJ-1 and MAP2 staining in NPC-derived differentiated neurons (Supplementary Fig. 4c).

In order to trace the effects that COSMOC knockout could have on the transition from pluripotency to neural/neuronal stages, we next used the CRISPR/Cas9 technology and successfully derived two different knockout (COSMOC^−/−^) hiPSC lines (Supplementary Fig. 5d, e). COSMOC^−/−^ hiPSCs and their respective COSMOC^+/+^ isogenic control line were differentiated into NPCs and we examined the expression of a small subset of neural progenitor associated genes (i.e., *VIMENTIN, SOX2, NESTIN*). Overall, Supplementary Fig. 5f–h show that the selected genes followed a similar trend of expression upon neural induction, which indicates that knocking-out COSMOC is not sufficient to impair the generation of neural progenitors. However, we cannot rule out the possibility that those NPCs are not phenotypically identic to the ones derived from COSMOC^+/+^ iPSCs.

### COSMOC impair synaptogenesis *via* PTBP2, a polypyrimidine tract binding protein

**Fig. 4:**
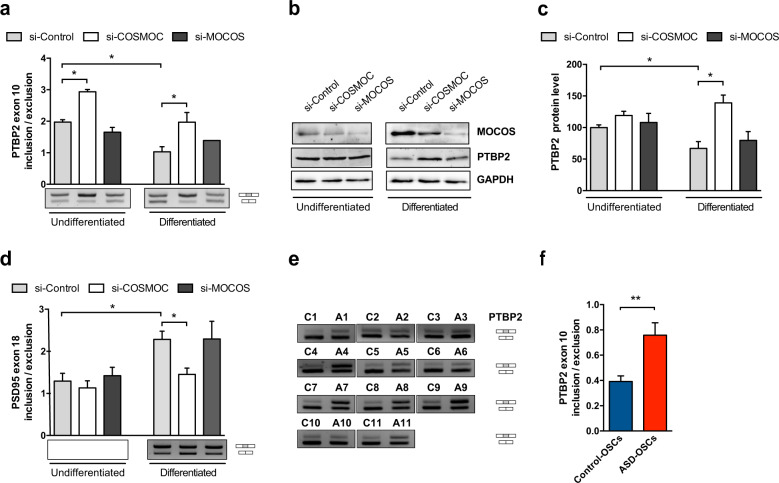
COSMOC downreguation alters *PTBP2* splicing in SH-SY5Y and stem cells from ASD patients. **a** Semi-quantitative RT-PCR analysis of *PTBP2* exon 10 splicing in undifferentiated or differentiated SH-SY5Y cells transfected with siRNAs (*n* = 4; Mann–Whitney test: *p < 0.05). **b** Representative western blot of MOCOS and PTBP2 expression and **c** quantification of PTBP2 protein level at 5DIV in undifferentiated and differentiated neuroblastoma cells. Cells were depleted in COSMOC or MOCOS expression and compared with cells treated with control siRNA. GAPDH was used as control. (*n* = 4; Mann–Whitney test: *p < 0.05). **d** Semi-quantitative RT-PCR analysis of *PSD95* exon 18 splicing in undifferentiated or differentiated SH-SY5Y cells treated with siRNAs and assessed at 5DIV. (*n* = 4; Mann–Whitney test: *p < 0.05). **e** Agarose gels and **f** quantification of RT-PCR products of *PTBP2* exon 10 inclusion. RNA was isolated from OSCs of ASD patients (from A1 to A11) and OSCs of their matched healthy individuals (from C1 to C11) (*n* = 11; Mann–Whitney test: **p < 0.01). Data are represented as mean ± SEM. For all graphs, *n* stands for the number of biological replicates of SH-SY5Y cells or OSC.

To determine potential changes resulting from *COSMOC* downregulation during SH-SY5Y differentiation, we examined the dynamic transcription pattern of genes selected from the aforementioned microarray data. Interestingly, a significant increase of *PTBP2* expression was observed throughout the time course of differentiation of SH-SY5Y depleted for *COSMOC* (Supplementary Fig. 6). Since the introduction of a premature termination codon in *PTBP2* transcripts results in their degradation *via* nonsense-mediated decay (NMD) [24], we examined the ratio of inclusion/exclusion of exon 10 (unspliced/spliced mRNA) in undifferentiated *versus* differentiated *COSMOC*-downregulated SH-SY5Y. Figure 4a shows a significant increase of *PTBP2* exon 10 inclusion and Fig. 4b, c indicate a nearly 50% increased expression of PTBP2 protein level when si-COSMOC-treated cells were compared with control cells at day 5 of differentiation. In a similar way, a substantial rise of exon 10 inclusion was noticed in *COSMOC*-targeted OSCs (Supplementary Figure 6a). Of note, PTBP2 was never disturbed in si-MOCOS treated cells (Fig. 2e, Fig. 4a–c and Supplementary Fig. 6a) and PTBP1 expression, a splicing regulator of PTBP2 [24], did not changed during neuronal differentiation of si-COSMOC–treated cells (Supplementary Fig. 6b). These data demonstrate that *COSMOC* downregulation induces the increase of PTBP2. This may occur directly or indirectly by either impairing PTBP2 autoregulation or disturbing the trans-splicing activity of its upstream RNA-binding regulators.

Of major interest, PTBP2 controls a genetic program crucial for neuronal maturation that regulates a variety of target genes including GABA receptors but also Gephyrin and PSD95 (Postsynaptic Density Protein 95), representing pivotal proteins for synaptic maturation and plasticity [25, 26]. Since PTBP2 may inhibit the inclusion of *PSD95* exon 18 [24], we decided to assess exon 18 splicing in *COSMOC*-downregulated SH-SY5Y cells ongoing differentiation. As shown on Fig. 4d, although no difference was observed in undifferentiated cell, knocking down *COSMOC* significantly decreased exon 18 inclusion (about 40%) in differentiated cells at Day 5. Altogether, these data support the notion that *COSMOC* deregulation may impair synapse development. Conversely, *MOCOS* knockdown failed to i) interfere with normal development of SH-SY5Y cells and ii) perturb *COSMOC* potential targets, supporting further an uncoordinated regulation of the transcriptional bidirectional unit, during neuronal differentiation (Fig. 4a–d, Supplementary Fig. 6a).

Taking this into account, and leveraging on our COSMOC^−/−^ iPSC-derived NPC model, we sought for possible alteration of PSD95 expression in COSMOC^−/−^ NPCs ongoing neuronal maturation. We found that PSD95 expression was rapidly detectable in COSMOC^+/+^ iPSC-derived neurons and stable at the two different time points we analyzed (Day 7 and Day 14 of differentiation). Surprisingly, PSD95 expression was found unstable in COSMOC^−/−^ neurons ongoing differentiation, indicating that PSD95 was changing in a random manner across the differentiation. These data support a possible role of COSMOC in the regulation/stabilization of PSD95 during neuronal maturation, and the absence of COSMOC may lead to defects associated to PSD95 expression (Supplementary Fig. 4i, j).

Finally, we assessed *PTBP2* expression in ASD patients. We observed that the unspliced isoform of *PTBP2* was upregulated in all ASD patients’ OSCs (Fig. 4e, f). These observations suggest that, in low *COSMOC*-expressing neuronal cells deregulation of PTBP2 and synapse-associated genes may alter neurotransmission and induce neurological disorders, including ASD.

## Discussion

The current study, built on our previous data that highlighted the reduced expression of MOCOS as a risk factor for ASD [8], reports an enthralling finding: *COSMOC*, an upstream antisense lncRNA located in the promoter region of *MOCOS*, is under-expressed in nearly all ASD patients of our cohort, the only exception being a patient affected by Asperger syndrome. Such a finding indicates that the deregulation of the *COSMOC*/*MOCOS* duet might be considered as a biomarker for ASD.

Although a set of candidate ASD-associated lncRNAs have been identified in co-expression network analysis [27], complex bidirectional transcription units have not been probed and divergent gene pairs still need to be carefully defined and characterized to help elucidate the genetic pathogenesis of ASD. It is therefore essential to further study the complex mechanisms of action of the COSMOC/*MOCOS* gene pair in the overall neurodevelopmental processes. However, while several physiological functions have been assigned to MOCOS, nothing was previously known about *COSMOC*. We show here that *COSMOC* and *MOCOS* display a similar pattern of expression. Nonetheless, the pair relationships are not fully bi-directional: *COSMOC* depletion induces a 50% reduced expression of *MOCOS* in OSCs but the reverse is not true. Although further research is needed to definitely exclude the presence of enhancer-like elements in the *MOCOS* promoter region and establish open chromatin state on this locus, our results indicate that COSMOC is finely tuned, could be a critical player in processes controlling neuronal survival and participate to neurodevelopmental processes.

At that point, our experimental approaches and results do not allow us to clearly define the mechanism(s) by which COSMOC regulates MOCOS. Actually, COSMOC function(s) may heavily depend on its subcellular localization and the adoption of specific structural modules with interacting partners [28]. In the nucleus, COSMOC may take part in the nuclear organization, mediating epigenetic changes to specific genomic loci and affecting the expression of its neighboring *MOCOS* transcripts *in cis* by recruiting transcription factors, RNA-binding proteins and chromatin-remodeling machineries to the site of transcription. This locus-specific environment could facilitate establishment of proper chromatin architecture to regulate *MOCOS* expression. Such a mechanism would be in agreement with our results and several reports investigating GC-rich regions and lncRNA functions [9, 29]. In the same way, the preferential localization and the role(s) of COSMOC in the cytoplasm are not completely resolved but modulation of key steps in post-transcriptional regulation of gene expression from mRNA processing to mRNA splicing, stability and translation or storage could also depend on cytoplasmic COSMOC.

In order to get a first overall picture of COSMOC primary and secondary targets, we performed a transcriptomic study based on the silencing of this lncRNA in OSCs. An impressive list of 639 genes is now associated to *COSMOC* depletion in OSCs, which could probably be extended to many other cell types of an organism. Reassuringly, eleven of them - *ALDH9A1*, *DHCR7*, *ENPP2*, *FABP3*, *GLO1*, *GLUL*, *LIPA*, *MOCOS*, *PPT1*, *PTBP2*, *SEMA5A* - were previously associated to ASD. Of note, among COSMOC-target genes, *PTBP2* and *SEMA5A*, but also *ATL3*, *KIS* or *YWHAH* are required for neural development, not to mention, *GLUL*, *PPT1*, but also *STX2*, *GNG12* and *FLOT1*, all involved in synaptic transmission in ASD (*1-5*; https://gene.sfari.org/). Interestingly, some genes (such as YWHAH and UHMK1) are genes with functional roles in neurodevelopment and other psychiatric diseases. These findings converge further to indicate that COSMOC could be part of the mechanism that regulates genes contributing to ASD. However, more works need to be done to define whether its deregulation is specific to ASD or could also be involved in other neurological disorders.

Interestingly, gene ontology indicates that *COSMOC*-regulated genes are strongly associated to lipid and sterol metabolism or cholesterol biosynthesis. These findings are in line with the reported associations between ASD symptoms and disorders of cholesterol biosynthesis, steroid anomalies and high prevalence of vitamin D deficiency [30, 31]. Indeed, pregnenolone, a cholesterol derivative, enhances the activation of emotion-controlling neuro-circuits and potentially relieves ASD symptoms [32, 33]. The cerebral cholesterol is essential for synapse functioning, myelin growth and axon wrapping, during early development [34]. Moreover, cholesterol modulates receptor binding and release of neurotransmitters such as oxytocin, GABA, acetylcholine and serotonin [30]. It also triggers the production of neurosteroids, a class of powerful modulators of GABA_A_ and glutamate receptors [35, 36]. Accordingly, it can be conjectured that *COSMOC* deregulation disturbs cholesterol biosynthesis and participates to ASD by altering neuronal physiology during development, when most cholesterol is derived from de novo synthesis by neurons and massive amounts of cholesterol are needed for growth [31, 36].

In addition, detailed inspection of the transcriptome revealed that the “Oxidation-reduction process” was presenting a significant number of deregulated genes (i.e., 46), indicating an altered detoxification circuitry. This process appeared to be particularly remarkable since we previously described (i) a disturbed redox homeostasis in stem cells of the ASD patients composing our cohort; and (ii) a greater susceptibility of *MOCOS* knockout animals to reactive oxygen species (ROS) [8]. Accordingly, we now show increased resting ROS production in stem cells treated with siRNA against COSMOC. These results are strengthened by the increased expression of PTBP2, a multifunctional splicing factor known to promote a switch in the expression of glycolytic enzymes [37]. Altogether, these data indicate that ASD patients of our cohort display metabolic disorders and suggest that COSMOC might modulate redox metabolism in stem cells. These findings are unsurprising as ASD are associated with impairments in basic physiological processes such as redox homeostasis. Increased levels of ROS, advanced glycation end products (AGEs) and lowered levels of antioxidants (such as glutathione peroxidase, superoxide dismutase, reduced glutathione) are indeed frequently reported in ASD children [10, 38]. Moreover, clinical trials have also reported an improved behavior in ASD patients receiving antioxidant therapy [39]. ROS are certainly essential regulators of multitudes of normal physiological processes in neurons including cognition and memory. Since synaptic activity is known to produce ROS, *COSMOC* under-expression through excessive ROS production may be associated with decreased performance in cognitive function in ASD patients. In that regard, the enhancer-like activity of COSMOC on the *MOCOS* gene may help us to understand some cellular functions of COSMOC. Hence, an unbalanced stress response may occur at least in part through inactivation of XDH and AOX, two MOCOS-dependent enzymes involved in purine metabolism and ROS production. Noticeably, alteration of sulfur metabolism and anomalies in purine metabolism have been reported in autism [40, 41] while anti-purinergic therapy was found to be effective in alleviating ASD symptoms [42].

The reason for the burst of *COSMOC* expression during the differentiation process of iPSC to neural progenitors has not been addressed yet. Recent studies have shown that stem cell fate is partially regulated by ROS, which mediates the redox state of cells as a secondary messenger [43]. Similarly, the progressive increase of *COSMOC* expression during differentiation of SH-SY5Y neuroblastoma cells may also be related to the profound changes in antioxidant defences that have been reported to occur during SH-SY5Ydifferentiation to neuron-like phenotype [44]. Keeping in mind that COSMOC is involved in oxido-reduction processes, one could speculate that a transient expression of COSMOC may participate to the fate decision of stem cells. In addition, lipid metabolic pathways are important for neurogenesis and large amount of lipids is used to produce new membranes required upon proliferation and differentiation. Elucidating how COSMOC affects lipid metabolism and homeostasis may help unify our understanding of the complex autism disorders.

Our data also revealed the control of COSMOC on the splicing of PTBP2, a RNA-binding proteins essential in post-transcriptional regulatory events. This is of major interest as PTBP2 is primarily expressed in the brain and, plays a crucial role in a splicing program required for neuronal differentiation [25, 26]. As a matter of fact, PTBP2 binds to intronic polypyrimidine clusters in pre-mRNA molecules and participates to the assembly of other splicing-regulatory proteins. Its association with autism is based on two studies reporting indel and missense variants in ASD probands [4, 45]. Interestingly, beside PTBP2, key regulators of alternative splicing, including the RBFOX1 family of proteins and SR100 are linked to neurodevelopmental disorders, including autism [2, 15]. In addition, alterations in the noncoding transcriptome and splicing of activity-dependent neuronal genes were reported in the cortex of ASD patients [15]. Supporting this, our data indicate that PSD95 expression, an important regulator of synaptic structure and plasticity, is affected by COSMOC, which may probably mediate through PTBP2 splicing defects. Altogether, the action of COSMOC on PTBP2 may play a wider action on numerous key cellular processes.

ASD are highly heritable with a number of risk loci ranging from hundreds to >1000. However, at best, each of them explains less than 1% of the cases and the vast majority of ASD cases are idiopathic. We report here on the identification of a new lncRNA, COSMOC, and its under-expression in nearly all autistic patients of our cohort. Misexpression of *COSMOC* and its adjacent partner *MOCOS* that is also found deregulated in most (80%) patients of our cohort [8], may be one of the common mechanisms contributing to numerous ASD cases and part of the dysfunctions observed in ASD. Collectively and/or separately, the *COSMOC/MOCOS* duo is involved in energy metabolism, neuronal maturation, synapse formation and neurotransmitter secretion, features strongly associated to neurodevelopmental disorders, including ASD. Its perturbed expression might as well trigger some of the ASD comorbid pathologies, notably the gastro-intestinal symptoms. Even though further studies are required to identify *COSMOC* interacting partners and the mechanisms underlying its action on the nervous system, this study reports on the first discovery of COSMOC and its importance to push forward investigation on its role in ASD.

## Supplementary information

Supplementary information
