# Supplementary material for: Impaired expression of the COSMOC/MOCOS gene unit in ASD patient stem cells
Source: Mol Psychiatry. 2020 Apr 23;26(5):1606–18. doi: 10.1038/s41380-020-0728-2 (PMC8159765; doi:10.1038/s41380-020-0728-2)
Supplement: Supplementary file 1 — Supplementary information [file 41380_2020_728_MOESM1_ESM.pdf]

Supplementary information

Impaired expression of the COSMOC/*MOCOS* gene unit in ASD patient stem cells

P. Rontani, O. Perche, L. Greetham, N. Jullien, F, B. Gepner, F. Féron, E. Nivet and M. Erard-Garcia*

Supplementary Methods, 6 Supplemental Figures and Figure Legends, 3 Supplemental Tables

SUPPLEMENTARY METHODS

ASD patients and Cell cultures

A complete written and oral information on the goal and procedure of this research was provided to the participants or their legal tutors and a signed informed consent was obtained from all of them before their involvement in the study. All procedures were approved by the local committee (Comité de Protection des Personnes, files#205016 and #205017) of Marseille.For ASD patients, nasal biopsies were performed by an ENT surgeon requiring a general anesthesia.

Human nasal olfactory stem cells (OSC) from 11 patients and 11 age- and gender-matched control individuals without diagnosed neuropsychiatric disorders were isolated and primary cultures were established as previously described (*1*). Cells were used at the same and low passage number and were regularly controlled as free of mycoplasma contamination throughout the study. Primary OSCs as well as SH-SY5Y, U138-MG, HepG2, HEK-293 and Caco-2 cell lines were cultured with DMEM high glucose media containing Glutamax media (Life Technologies) and supplemented with 10% fetal bovine serum and 1% penicillin/streptomycin. THP-1 cells were cultured with RPMI media (Life Technologies), 10% fetal bovine serum and 1% penicillin/streptomycin. Cells were incubated at 37°C in a humidified chamber containing 5% CO_2. Culture media was changed every three days and cells were passaged with Trypsin/EDTA (Life Technologies) when they reached ~90% confluency.

For induced pluripotent stem cells (iPSCs) culture, OSCs from two control individuals were reprogrammed by using a retroviral-based reprogramming strategy. In short, cells were infected with an equal ratio of retroviruses encoding for Oct3/4, SOX2, Klf4 and c-MYC by spinfection of the cells at 1,850 rpm for 1 hour at 32°C in the presence of polybrene (4 mg/ml). After two

serial infections, cells were passaged onto fresh irradiated mouse embryonic fibroblasts (iMEFs, amsbio) and switched to a medium containing DMEM/F12 (Life Technologies) supplemented with 20% Knockout Serum Replacement (Life Technologies), 1 mM L-glutamine (Life Technologies), 0.1 mM non-essential amino acids (Life Technologies), 55 mM b-mercaptoethanol and 10 ng/ml bFGF (PeproTech). For the derivation of hiPSC lines, iPS-like colonies were manually picked and maintained on fresh iMEF feeder layers for four to five passages before being transferred onto Matrigel/mTesR1 conditions. iPSCs features were confirmed by microscopic observation and only colonies displaying typical iPSC-like colony morphology were selected and further confirmed by immunocytochemical analyses showing the expression of typical markers (Oct3/4, SOX2, NANOG, SSEA4, TRA1-60 and TRA1-81). Pluripotency was also confirmed by the capacity of the chosen iPSC lines to give rise to all three germ layers under differentiating conditions. For this study, 2 validated iPSC clones were randomly chosen (one from each donor) and transferred into feeder-free conditions. In brief, human iPSCs were cultured in chemically defined growth media, StemMACS™ iPS-Brew XF medium (Miltenyi Biotec), on growth-factor-reduced Matrigel (BD Biosciences)-coated plates. For iPSC maintenance/amplification, 70–80% confluent human iPSCs were treated with with an enzyme-free solution (hereafter referred as Gentle Dissociation Solution) and containing 0.5 mM EDTA (Life Technologies), D-PBS (Life Technologies) and 1.8 mg/mL NaCl (Sigma). iPSCs were incubated for 2 min in Gentle Dissociation Solution at 37°C and the colonies were dispersed to small clusters and lifted carefully using a 5 mL glass pipette at a ratio of 1:4. When necessary, differentiated areas were removed from iPSC cultures prior to passaging in order to maintain the cultures in undifferentiated state prior to proceeding to their

differentiation. Both iPSC lines were maintained in an incubator (37°C, 5% CO_2) with medium changes every day.

Derivation of NPCs from iPSCs

Neural progenitor cell (NPC) were differentiated from hiPSCs by applying two different differentiation protocols. 1) Induction of NPCs was based on previous reports with slight modifications (*2, 3*). One day before induction, hiPSCs were passaged onto growth-factor reduced matrigel (Corning)-coated plates and maintained in stemMACS™iPSC-Brew XF medium (Mylteni Biotec) overnight. At day 0, culture medium was then replaced with Neural Induction Medium 1 (NIM-1: 50% DMEM/F12 (Invitrogen), 50% Neurobasal (Invitrogen), 1×N2 (Invitrogen), 1×B27 (Invitrogen), 10ng/ml hLIF (Prospec), 4 μM CHIR99021 (Reagents Direct), 3 μM SB431542 (Stemgent), 2 μM Dorsomorphin (Sigma) and 0.1 μM Compound E (EMD Chemicals Inc.). Cells were treated with NIM-1 for 2 days, and then switched for another 5 days to Neural Induction Medium 2 (NIM-2: 50% DMEM/F12, 50% Neurobasal, 1×N2, 1× B27, 10 ng/ml hLIF, 4 μM CHIR99021, 3 μM SB431542 and 0.1 μM Compound E). The cultures were then passaged onto growth-factor-reduced matrigel-coated plates with Accumax (Life Technologies) and cultured in Neural Stem cell Maintenance Medium (NSMM) containing 50% Advanced DMEM/F12, 50% Neurobasal, 1x N2, 1x B27, 2 mM GlutaMAX, 10 ng/mL hLIF, 3 μM CHIR99021, and 2 μM SB431542. Generated NPCs with this protocol were maintained on growth-factor-reduced matrigel in NSMM. NPCs were passaged once reaching 80% confluency using Accumax and seeded at about 20% confluency. Medium was changed every day. For the initial 3 passages, NPCs were treated with 10 μM Y-27632 (TOCRIS) during splitting. 2) Induction of NPCs was alternatively performed following the monolayer culture method with the

STEMdiffTM SMADi Neural Induction Kit (Stem Cell Technologies) following manufacturer's instructions with slight modifications. In short, undifferentiated cultures of hiPSCs were treated with Gentle Dissociation Solution for 4 min, then the solution was removed and cells incubated in Accumax for 4 min. After this, iPSCs were dislodged as single cells and transferred into Dulbecco's Modified Eagle Medium (DMEM)-F12 + 20% KnockOut Serum Replacement (Life Technologies) with a minimum of 10 times the volume of Accumax used for single Cell dissociation. Then, we centrifuged cells at 200×G for 5 min. Cells were resuspended on D-PBS, counted and centrifuged at 200×G for 5 min. Last, cells were resuspended in STEMdiffTM SMADi Neural Induction Kit + 10 µM µM Y-27632 and plated onto growth-factor-reduced-coated plates (320,000 cells cm−2). Cells were maintained in an incubator (37°C, 5% CO_2) with medium changes every day and observed under a microscope on a daily basis. Between 6 and 9 days after induction, cells were harvested as single cells using Accumax, transferred into DMEM-F12 media, counted and centrifuged at 200×G for 5 min prior to be resuspended in STEMdiffTM SMADi Neural Induction Kit + 10 µM µM Y-27632 and plated onto growth-factor-reduced-coated plates (270,000 cells cm−2). Cells were maintained in an incubator (37°C, 5% CO_2) with medium changes every day and observed under a microscope on a daily basis. 5 days post-seeding, cells were once more passaged following the same procedure. Finally, 5 days after the last passage (i.e. 16 to 19 days post-induction), differentiated cells were harvested as single cells using Accumax, transferred into DMEM-F12 media, counted and centrifuged at 200×G for 5 min prior to be resuspended in STEMdiffTM Neural Progenitor Medium (Stem Cell Technologies) and seeded onto growth-factor-reduced matrigel-coated plates (125,000 cells cm−2). iPSC-NPCs were maintained in an incubator (37°C, 5% CO2) with medium

changes every day and passaged with Accumax when reaching 80-90% confluency. Of note, iPSC-NPCs between Passage 3 and 5 were used for further maturation into neurons.

Differentiation of hiPSC-NPCs into neurons

hiPSC-NPCs were differentiated into mature neurons with the BrainPhys^{TM} Neuronal Medium Kit (Stem Cell Technologies) following manufacturer's instructions; which allows for the generation of mature neuronal cultures as previously described (*4*). In short, hiPSC-NPCs were harvested as single cells using Accumax, transferred into DMEM-F12 media, counted and centrifuged at 200×G for 5 min prior to be resuspended in supplemented BrainPhysTM Neuronal Medium (following manufacturer's recommendations) and plated onto Poly-L-Ornithin (10 µg/mL) /Laminin (4 µg/mL) -coated plates (50,000 cells cm^{-2}). Cells ongoing neuronal differentiation/maturation were maintained in an incubator (37°C, 5% CO_2) with half medium changes once a week. Cells were harvested at indicated time points throughout the study.

SH-SY5Y differentiation

SH-SY5Y were transferred onto 6-well or 24-well plate at a density of 5×10^4 and 1×10^4 cells/well respectively. Cell differentiation was performed in Neurobasal medium with 1% glutamine, 1% P/S, 1x B27 supplement (all Life Technologies), 50 ng/ml hBDNF (Miltenyi Biotec), 10 µM retinoic acid, 2mM dibutyryl AMPc, 20 mM KCl (all Sigma-Aldrich) as previously described (*5*). In contrast to currently used SH-SY5Y models, this improved model captures early neurodevelopmental processes with high fidelity. Differentiation medium was changed every day and cells were harvested at day 0, 1, 3, 5, 7 and 9 of differentiation in vitro (DIV) for RNA and/or protein analysis. For knockdown experiments, undifferentiated cells were

incubated during 24 hours with siRNA before the initiation of differentiation and the differentiation was stop at day 5.

Generation of CRISPR-Cas9 mediated COSMOC-Knock-Out hiPSCs

For CRISPR-Cas9 plasmids generation, we first modified the pSpCas9 (BB)-2A-Puro (PX459) V2.0 (Addgene plasmid # 62988) (*6*) in order to replace the Cbh promoter for the EF1 alpha promoter. In short, the pSpCas9 (BB)-2A-Puro (PX459) V2.0 was digested by XbaI+AgeI and the large band (8364bp) was purified by gel migration to recover the plasmid without the CBh promoter (810bp band). The Human EF1 alpha promoter was amplified from hIPS cells with primers pAB01 and pAB02
(5'-aattctgcagacaaatggctctagaGGCTCCGGTGCCCGTCAGTG-3' and
5'-tccttatagtccatggtggcaccggTCACGACACCTGAAATGGAAG-3' respectively, in which uppercases are nucleotides matching pEF1alpha) from the distal part (pAB01) to the proximal one (pAB02, reverse complement) (1188bp long). The lower case nucleotides match the pX459 backbone at the XbaI cut end (pAB01) and EagI cut end (pAB02).

The final product cAB03 (*i.e.*, pX459-pEF1alpha) was then obtained by SLIC method mixing the pX459 XbaI-EagI fragment and the EF1alpha PCR product as previously described (*7*). Therefore, cAB03 was used to produce two distinct plasmids, each containing a *COSMOC* specific single guide RNA (i.e., sgRNA containing crRNA + TracrRNA) sequence aiming at cutting a 140bp fragment portion within the exon1. To that end, CRISPR sgRNAs were designed using Crispor design tool (http://crispor.tefor.net) and cloned into cAB03 as previously described (*6*). For each of the two sgRNAs, a 20nt long *COSMOC* specific sequence was selected as follows: sgRNA1: GCTGGCCTGAAAGTGAAGAC; and sgRNA2: TTTAGCTATGTCTCGCGGAG. In short,

each of the two selected COSMOC specific sequences was cloned by BbsI-mediated digestion of cAB03 followed by ligation using cohesive-end cloning. Plasmids were transformed into DH5-alpha chemically-competent cells and individual clones were first screened by PCR to validate the insertion and ultimately validated by sequencing analysis.

For the generation of COSMOC-Knock-Out hiPSC lines, undifferentiated hiPSCs were treated with Gentle Dissociation Solution for 4min at Room Temperature (RT). The Gentle Dissociation Solution was then removed and cells were treated with Accutase (Life Technologies) for 4 additional minutes at RT. Cells were collected as single cells (*i.e.*, dissociated colonies) and transferred into a tube containing a solution of DMEM/F12 (Life Technologies) supplemented with 20% of Knock-Out Serum Replacement (Gibco), using a minimum of 5 times the volume of accutase used for cell dissociation. Cells were centrifuged at 200xg for 5min, the cell pellet was washed with D-PBS without Ca^{2+} and Mg^{2+} (Lonza). Cells were centrifuged one more time at 200xg for 5min. The cell pellet was then re-suspended with the Resuspension Buffer R (Neon® 100 µl Kit, ThermoFisher) to a final concentration of 10,000 cell/µl. 100 µl of the cell suspension was transferred to a sterile 1.5ml microcentrifuge tube and mixed with 4 µg of each of the two plasmids containing sgRNA1 and sgRNA2 respectively. Cells were then electroporated with the Neon Transfection System (Thermofisher) using the following parameters: 1100V, 30ms, 1 pulse. The electroporated cell suspension was then flushed into 1 well of a 6-well Matrigel-coated plate containing StemMACS™ iPS-Brew XF medium (Miltenyi Biotec) supplemented with 10 µM Y-27632 (TOCRIS). The next day, media was replaced with StemMACS™ iPS-Brew XF medium supplemented with Puromycin at 0,5µg/ml for 48 hours. Of note, one well containing cells electroprated without plasmids was used as control to define the best time

window at which the Puromycin treatment had to be stopped; *i.e.,* when all control cells were dead. Puromycin-resistant cells were maintained in culture with iPS-Brew medium and changed every second day until individual and clonal colonies could be observed. Each individual colonies were manually picked and cut in two halves: one half was used for genomic DNA extraction followed by PCR screening and the other half was slightly dissociated by 2-3 pipetting up and down (using a 200 µl pipette tip) and plated back onto one well of a 24-well Matrigel-coated plate containing StemMACS™ iPS-Brew XF medium (Miltenyi Biotec) supplemented with 10 µM Y-27632 (TOCRIS). Regarding the latter, each clone was maintained in culture until (in)-validation for the loss of the targeted portion of the gene of interest (COSMOC, expected ~ 140 nt loss in exon 1) as evaluated by PCR analysis. For genomic DNA extraction, the other half of the colony was transferred into a 0.2ml PCR tube and centrifuged 5min at 300xg. Then, the supernatant was removed and the pellet washed with 150µl D-PBS without Ca^{2+} and Mg^{2+} and centrifuged once again 5min at 300xg. The supernatant was then removed and the cell pellet re-suspended with a 20µl mix containing: 1X of the 5X PrimeSTAR GXL Buffer (Clontech), proteinase K [0.167mg/ml] and ddH_20. DNA extraction was performed using a thermocycler with the following settings: 3hours at 55ºC and 30min at 95ºC. Then, a 10 µl PCR reaction was performed using 1 µl of the extracted genomic DNA and 9 µl of a PCR mix (1X of the 5X PrimeSTAR GXL Buffer, PrimeSTAR GXL DNA polymerase [0.25 U/10 µl], dNTPs [200µM each], primer COSMOC seq F and R [0.3 µM each], see Supplementary Table 2, ddH_20) and PCR reaction was performed using the following PCR conditions: 1min at 94°C for one cycle, then 10sec at 98°C and 1min at 55°C for 35 cycles and to finish, 5min at 68°C for one cycle. Then, the PCR product was run on a 2% agarose gel electrophoresis to analyze the size of the amplicon for each individual clone (expected profiles: 809bp (Wild Type); ~699bp (Knock-Out

presenting a 140bp deletion); two bands at 809bp and ~699bp (heterozygous lines)). Only those clones that were effectively Knock-Out for COSMOC were amplified.

Transfection of cells and Primers

Twenty-four hours after seeding, cells were transfected with Stealth RNAi™ siRNAs (Life Technologies) using Lipofectamine RNAimax (Life Technologies). Experiments using Stealth RNAi™ siRNA negative controls (Life Technologies) were performed in parallel. The primers sequences were as follows:

si*MOCOS*: 5'-GAGCCAGCTCGAAAGCTTCACTAGT-3';

siCOSMOC 146: 5'-TTGAACTTGGCATTCCAAGG-3';

siCOSMOC 489: 5'-TTTTCTTTCTGGTTTACGCAAG-3';

siAOX1: 5'-AGGCCAGATTGAAGGTGCATTTATT-3';

siXDH: 5'-GAACACCATGAAGACCCAGAGCTTT-3'.

For RNA extraction, transfected stem cells were always harvested48 hours after siRNA transfection. For proteins quantification, they were harvested at 48 and 72 hours after transfection.

RNA preparation, PCR and qPCR

Total RNA was extracted using TRIzol reagent (Invitrogen) according to the manufacturer's instruction. Cytoplasmic/nuclear RNAs were fractioned and extracted using PARIS™ KIT (ThermoFisher Scientific). RNAs were reverse transcribed by M-MLV reverse transcriptase (Invitrogen) with random hexamers and PCR reactions were run with Taq DNA Polymerase (Invitrogen). Primers sequences are reported in Supplementary Table S2.

Quantitative PCR experiments were carried out with the 7500 Fast Real-Time PCR system (ThermoFisher Scientific), using TaqMan™ Fast Universal PCR Master Mix and TaqMan™ Gene Expression Assays (ThermoFischer Scientific). Assays ID are reported in Supplementary Table S3.

Oxidative stress induction

OSCs were cultured at a density of 2 x 10^6 cell/well onto 6-well plate. Twenty-four hours after seeding, cells were stressed for 2 or 4 hours in the presence of H_2O_2 (500µM) before RNA extraction.

ROS assay and flow cytometry analysis

Forty-eight hours after siRNAs transfection, intracellular ROS was measured by staining with CellRox® Deep Red Flow Cytometry Assay kit (Life Technologies). Stem cells were harvested and stressed with tert-butyl hydroperoxide (TBHP; 200 µM) at 37°C for 30 minutes. Cells were then treated with CellROX reagent (500 nM) at 37°C for 45 minutes. Staining was measured using FacsCanto cytometer (Becton Dickinson) and data were analyzed using FACS DIVA software.

Western blot

Protein concentrations were quantified using the Bio-Rad DC™ protein assay kit following manufacturer's instructions. Protein extracts (30 µg) were separated on 16,5% Tris-Tricine gel for cleaved Caspase 3 and 10% SDS-PAGE gel for other proteins. Then they are transferred to nitrocellulose membranes (GE Healthcare). After blocking, membranes were incubated with

rabbit anti-MOCOS (Novus Biologicals, NBP2-14243, 1/100), rabbit anti-cleaved caspase 3 (Cell Signaling, 9664, 1/1000), rabbit anti-PTBP2 (nPTB-IS2 4860, 1/1000), goat anti-PTBP1 (Novus Biologicals, NB100-1310, 1/1500) or mouse anti-GAPDH (Millipore, MAB374, 1/10000) antibodies overnight at 4°C, and the appropriate horseradish peroxidase-conjugated secondary IgG antibodies (Jackson ImmunoResearch) for 2 hours at room temperature. Signals were visualized with ECL chemiluminescence kit (GE Healthcare) and quantified using ImageJ software.

Immunocytochemistry

Cells were fixed in 4% paraformaldehyde for 15 minutes at room temperature before immunostaining. After blocking with PBS 3% BSA, 0.1% Triton X, cells were incubated overnight at 4°C with the following primary antibodies: chicken anti-MAP2 (Abcam, Ab5392, 1/300), mouse anti-β-3-tubulin (Sigma-Aldrich, T8660, 1/300), rabbit anti-SOX2 (Stemcell, 60055, 1/200), rabbit anti-PAX6 (Biolegend, 901301, 1/300) or mouse anti-nestin (Temecula, MAB5326, 1/500). The corresponding secondary antibodies [coupled to Alexa Fluor® 488 or 594 (Molecular Probes, 1/800)] and Hoechst (Sigma-Aldrich, 33342, 1/1000) for 2 hours at room temperature. Cells were examined using a Zeiss LSM 710 Laser Scanning Confocal Microscope (Zeiss).

Microarray gene expression

Stem cells from two different healthy individuals were collected 48 hours after treatment with siRNAs. RNA was extracted as described above. Genome-wide transcriptional profiling was performed by Human Exon 1.0 ST arrays (Affymetrix) following manufacturer's instructions.

Each condition was performed in duplicate. GeneChip CEL files were analyzed using GeneSpring (Agilent Technologies). The log2 values were obtained and the microarray data were normalized using RMA16 algorithm. Statistical data analyses were done on all samples for each group. Similarly, enrichment rankings are based on all samples per group. Data were filtered with GeneSpring analysis using a cutoff of at least 1.3× up or down (Fold change 1.3, FC1.3). Subsequently, one-way ANOVA was performed on a filtered list. The Benjamini–Hochberg false discovery rate (FDR) was set to 5%, and the P value was set to < 0.05 (ANOVA test). The Database for Annotation, Visualization, and Integrated Discovery (DAVID) was used to find gene ontology-enriched terms.

SUPPLEMENTARY FIGURES AND FIGURE LEGENDS

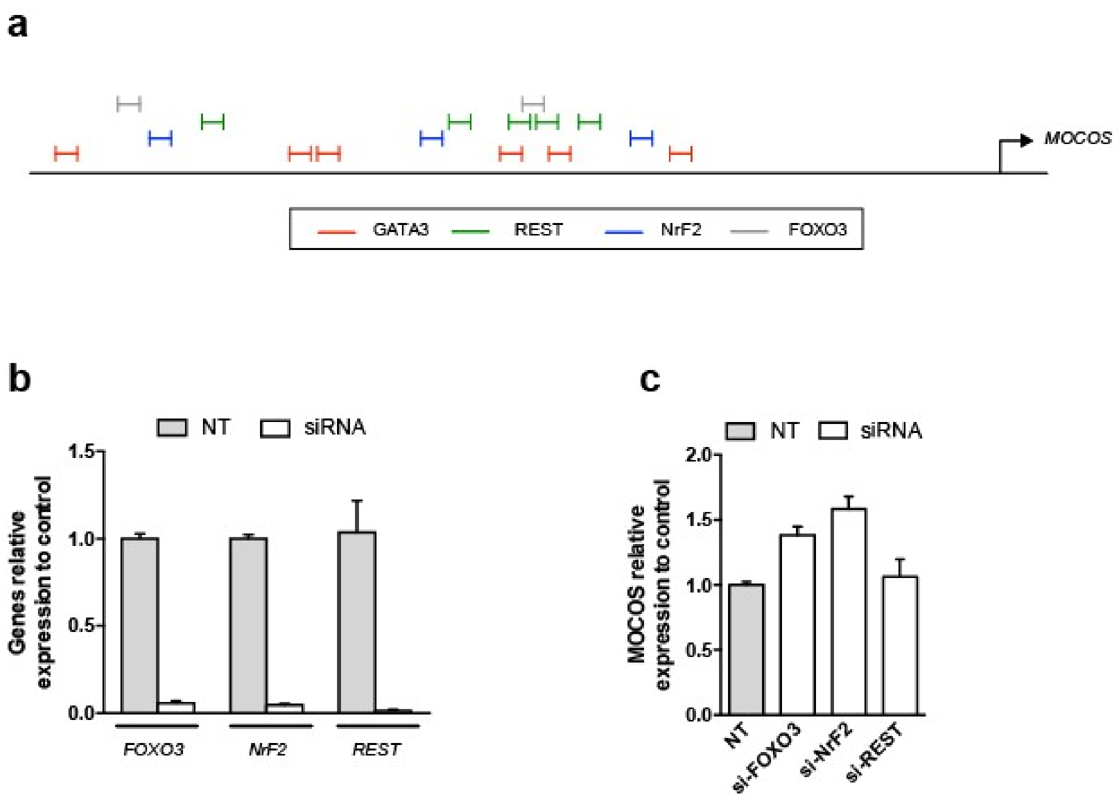

Supplementary Figure 1.Prediction and experimental assessment of transcription factor binding sites in the 1 kB region upstream of the major transcription start site (TSS) of the *MOCOS* gene.

(a) Mapping of putative transcription factors biding sites in the human MOCOS promoter region (not to scale, using MatInspector (Genomatix) and TFBIND software). Sequences, positions from TSS and similarity scores are respectively as follow: GATA3 biding sites: (GAGAGAGCG; 1551-1541; 0.839167), (AGTTATCTC; 1140-1131; 0.867966), (CACCATCCC; 1094-1085; 0.880372), (GGGATTGAA; 995-804; 0.845370), (TAGAGAGGG; 729-720; 0.834293), (CCTAATCTT; 514-505; 0,920691); REST biding sites (GCAGCTGGCCTGGATGCAGCA;

1283-1262; 0,732041), (CCCAGCAGCGGGTTCCGCACC; 1018-997; 0.728069), (CGCTGTGTCCCAGGTGCTCTG; 841- 820; 0.716505); NrF2 binding sites: (GGCGGGAGAG; 1394-1384; 0.812500) (CACTTCCCGC; 905-895; 0.844234) (CTCTTGCTTT; 741-731; 0.808140) (GCGGGAAGTG; 703-793; 0.844234) (GCAGGAAGTA; 662-652; 0.811773) and FOXO3 biding sites: (CTTAGTGG; 1435-1427), (CTTATTCC 786-778). (**b**) RT-qPCR analysis of REST, NrF2 and FOXO3 expression in stem cells transfected with siRNA against FOXO3, NrF2 and REST respectively and compared to control cells. GATA3 knockdown was not assessed because it is lowly expressed in stem cells. **(c)** RT-qPCR analysis of *MOCOS* expression in stem cells depleted in FOXO3, NrF2 or REST (n=3). Data are represented as mean ± SEM.

a

5'-CCAGCCTCCCAGTCTCTCGGAATCCGCAGCCTAGGTGTGGCGCCCCGACCGGACTTTCACTTCTGGCCAGCCCTTTCCCCAC
CTGGGCGCGGGAGCGGGTGCCAGTCTTTAAACAACCTCTCGATGGGTCCCACGAAGATGTTTCCAGACCCTTGGAATGCCAAGT
TCAAGTTTAGCTATGTCTCGCGGAGAGGCCGGTGGAAGAAGCAACGAGAATGAAGCACCCCAGTTCTCTGCTGAGCACATGGGC
ATCTGCAATAAAGATTTAATTTCCCAGCTTCTCCTGAAGCTCGGTATGGCCACAACACTAAATTCTGCCCGAGGAGATTAAGCAAAAT
AGTATGGGACTTCCAAGAAATGTTTTTAAAGTCAGGGGCAGGCCTTTCTTCATGCCTTCTTCCTCTTTGCTGGCTGGAACGCAAAG
ACCATGGCAGGAGGCCAAGCAGCCATCCTGGAAGGTGAAAGCCTCATACTAAGGACGTCAGACAGCGAAATAAGAGCCTGGGTC
CTTGACCCTGTAGACATCTCCCTCCCCATCCTGGTCTGTCTGCCTTGACTCCTTTCATATGAGAGAAATAAACTTTTAACTTGCGTAA
ACCAGAAAGAAAA-3'

b

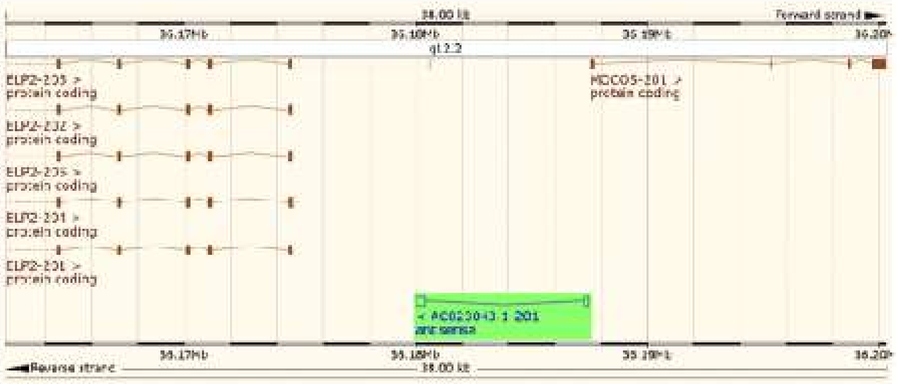

c

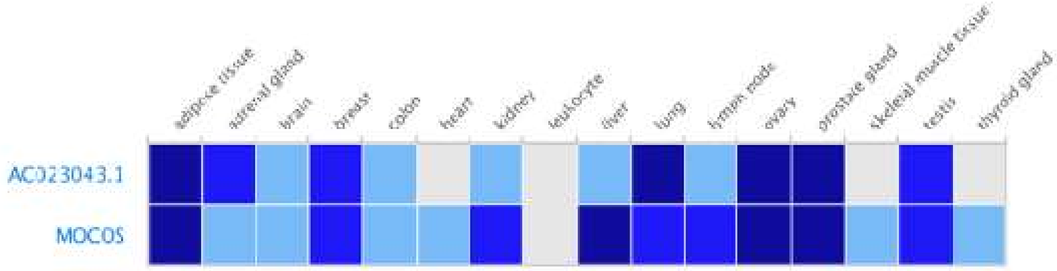

d

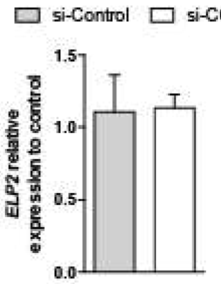

Supplementary Figure 2. Structure, localization and expression of COSMOC.

(a) Nucleotide sequence of COSMOC: the first and the second exons are written in green and blue respectively. **(b)** Localization of COSMOC (noted AC023043.1), *MOCOS*, and different variants of ELP2 on the human genome (Ensembl). (**c**) COSMOC and *MOCOS* mRNA expression in human tissues (EMBL expression atlas, Illumina body map data). **(d)** RT-qPCR analysis of *ELP2* expression in stem cells depleted in COSMOC expression (n= 3). Data are presented as mean ± SEM.

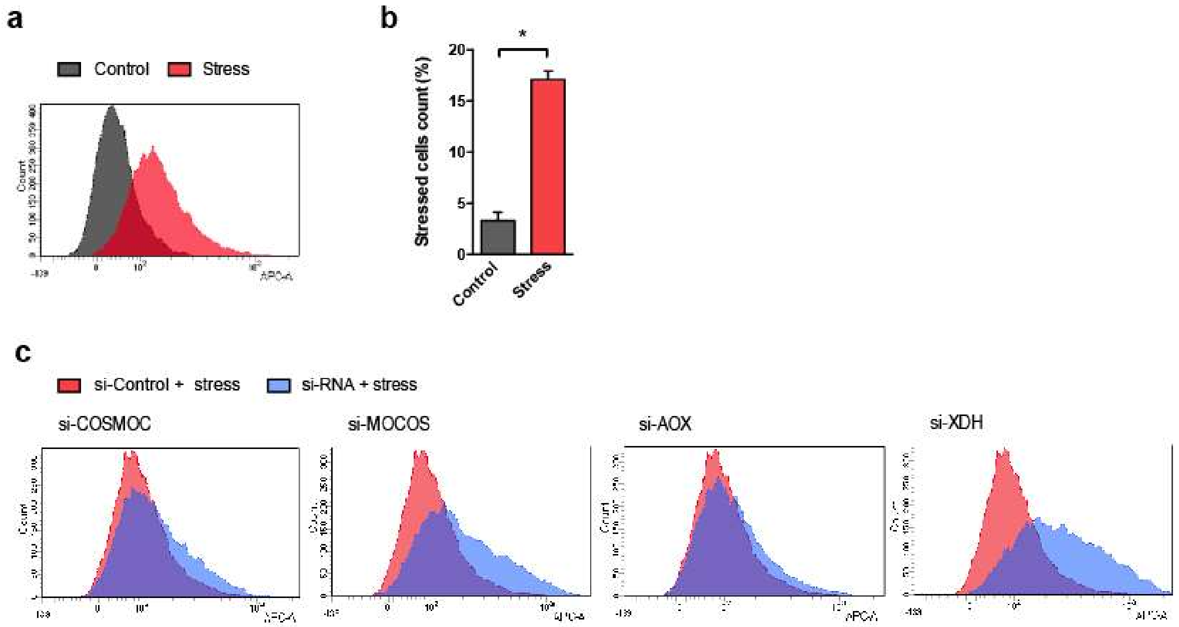

Supplementary Figure 3. Stress induction in olfactory stem cells depleted in COSMOC, MOCOS and MOCOS-dependent genes.

(a) Flow cytometric detection of reactive oxygen species (ROS) and (**b**) quantification of stressed cells in control and stress conditions (n=4; *p < 0.05; Mann-Whitney test). **(c)** Flow cytometric detection of ROS in stem cells after siRNA treatment and stress induction. Data are presented as mean ± SEM.

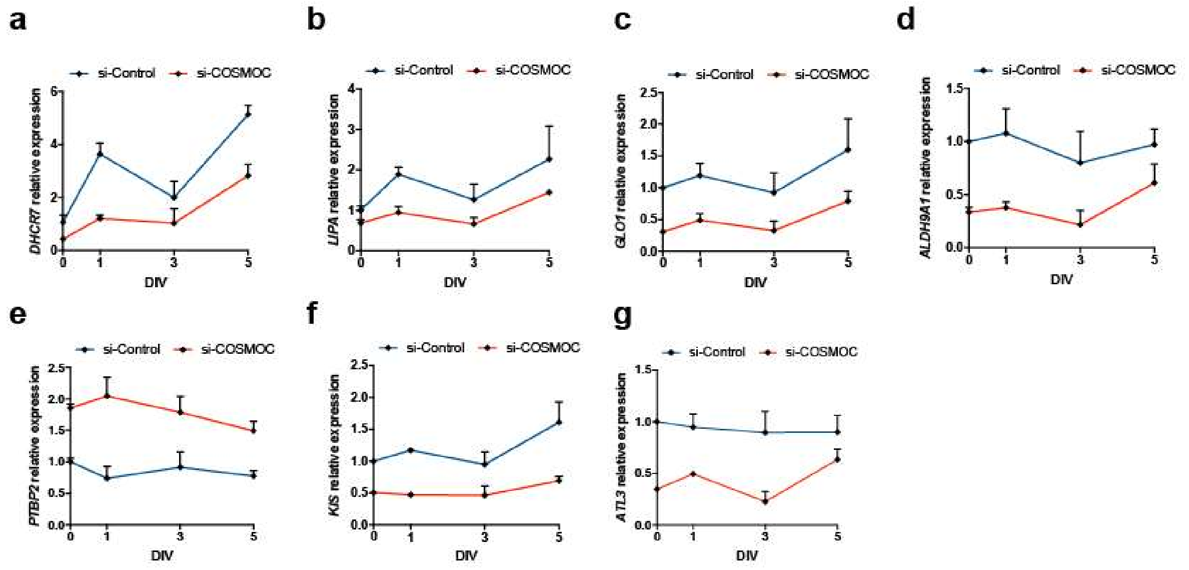

Supplementary Figure 4. Expression of a selection of genes during neuronal differentiation of SH-SY5Y cells.

(a-g) RT-qPCR analysis of a variety of genes identified in the transcriptomic study and examined in SH-SY5Y cells depleted for COSMOC expression or control cells (n=3). These time-curves illustrate differences in expression level of genes involved in cholesterol biosynthesis process (such as DHCR7 and LIPA), redox homeostasis (GLO1 and ALDH9A1) and synaptic transmission or neural development (PTBP2, KIS and ATL3). Data are presented as mean ± SEM.

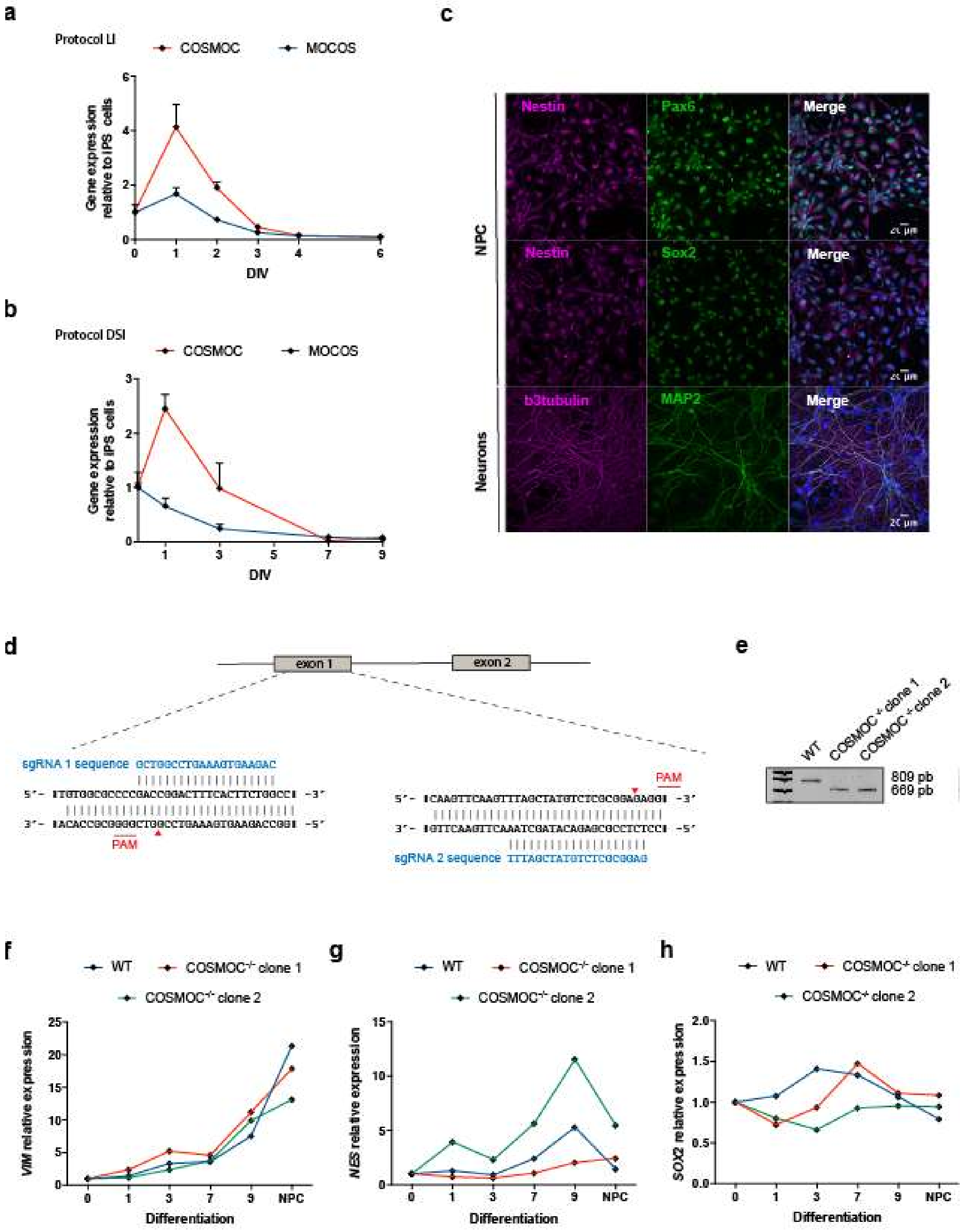
a
Protocol LI
COSMOC
MOCOS
Gene expression relative to IPS cells
DIV
b
Protocol DSI
COSMOC
MOCOS
Gene expression relative to IPS cells
DIV
c
NPC
Neurons
Nestin
Pax6
Merge
Nestin
Sox2
Merge
b3tubulin
MAP2
Merge
d
exon 1
exon 2
sgRNA 1 sequence GCTGGCCTGAAAGTGAAGAC
PAM
sgRNA 2 sequence TTTAGCTATGTCTCGCGGAG
e
WT
COSMOC-/- clone 1
COSMOC-/- clone 2
809 pb
669 pb
f
WT
COSMOC-/- clone 1
COSMOC-/- clone 2
VIM relative expression
Differentiation
g
WT
COSMOC-/- clone 1
COSMOC-/- clone 2
NES relative expression
Differentiation
h
WT
COSMOC-/- clone 1
COSMOC-/- clone 2
SOX2 relative expression
Differentiation

Supplementary Figure 5. ***COSMOC*** **expression during early neurodevelopmental processes.** **(a, b)** RT-qPCR analysis of COSMOC and *MOCOS* expression during differentiation of iPSC into Neural Progenitor Cells (NPC) using two different induction protocols Two validated iPSC clones were used in this study and gene expression was normalized to time-point 0 (n=3). (**c**) Immunocytochemistry showing effective neuronal differentiation with markers PAX6, SOX2 and NESTIN in differentiated hiPSC-derived NPCs and with the βIII tubulin and MAP2 markers in differentiated hiPSC derived neurons. **(d)** Schematic representation of the CRISPR/Cas9-based strategy used to invalidate COSMOC in human iPSCs. The genomic locations of gRNAs sequences targeting COSMOC and the Protospacer Adjacent Motif (PAM) are shown. The red triangles indicate cleavage sites. **(e)** Agarose gel of PCR products confirming COSMOC deletion in two different clones of hiPSC (clone 1 and 2) when compared with the size of the wild type product in the parental iPSC. **(f–h)-** RT-qPCR analysis of the neuronal differentiation markers Vimentin (**f**) Nestin (**G**) and SOX2 (**h**) during the progression of hiPSC clones to committed NPC. Gene expression was normalized to time-point 0 (n=3). Graphs show mean ± SEM.

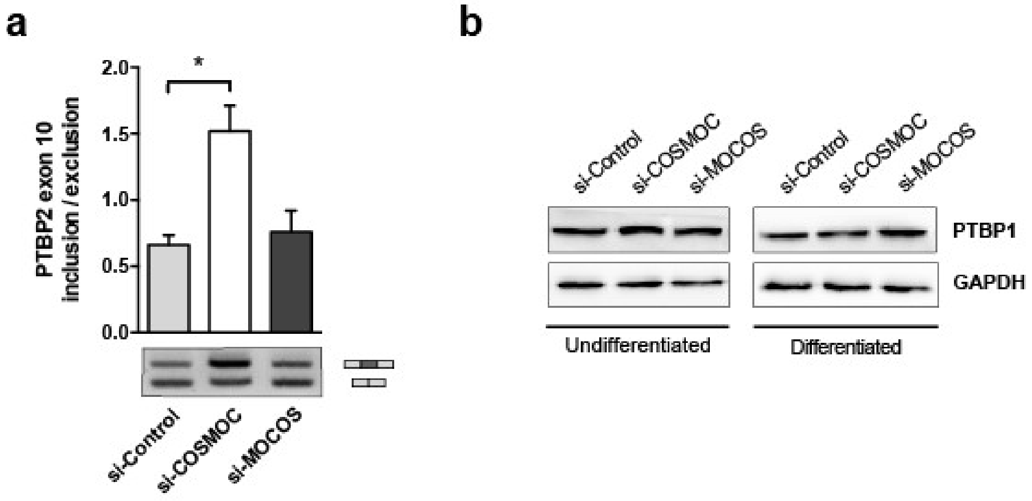

Supplementary Figure 6. PTBP2 exon 10 inclusion in adult stem cells and PTBP1 expression during differentiation of SH-SY5Y cells.

(a) Semi-quantitative RT-PCR analysis of PTBP2 exon 10 splicing in stem cells transfected with siRNAs against COSMOC or *MOCOS* and compared to cells transfected with scrambled siRNA for negative control (n=4*; $p < 0.05$; Mann-Whitney test). Data are represented as mean ± SEM.

(b) Representative western blot of PTBP1 expression in undifferentiated SH-SY5Y cells or cells differentiated for 5 days after transfection with siRNAs against COSMOC, *MOCOS* and compared to control (n=4; Mann-Whitney test).

Supplementary Table 1

Primers list:

Gene	Forward	Reverse
COSMOC	5′-CCTCCCAGTCTCTCGGAATC-3′	5′-TCTCGTTGCTTCTTCCACCG-3′
GAPDH	5′-CATCACCATCTTCCAGGAGC-3′	5′-GGATGATGTTCTGGAGAGCC-3′
MOCOS	5′-CACCACCGCAGAAGACTACAC-3′	5′-CACGTTCCGCATACCCACTAC-3′
PSD95	5'-ATGGCCGGGATTACCACTTTGT-3	5'-CTGTGAACTCCTGCTCCAGCTT-3'
PTBP2	5'-GGCAATACAGTCCTGTTGGTT-3'	5'-CCATCTGTATTAGAGCGCTGT-3'

Supplementary Table 2

TaqMan™ Gene Expression Assays ID

Gene	Assay ID
ABCA1	Hs01059137_m1
ACAT2	Hs00255067_m1
ALDH9A1	Hs00997881_m1
COSMOC	Hs01386774_m1
CDK1	Hs00938777_m1
DHCR7	Hs01023087_m1
ELP2	Hs01548550_m1
FOXO3	Hs00921424_m1
GLO1	Hs00198702_m1
INSIG1	Hs00356479_g1
LIPA	Hs01548815_m1
MALAT1	Hs00273907_s1
MAPT	Hs00902194_m1
MOCOS	Hs00215742_m1
NANOG	Hs04260366_g1
NFE2L2	Hs00975961_g1
PAX6	Hs01088114_m1
PTBP2	Hs01082109_m1
REST	Hs05028212_s1
SIRT1	Hs01009005_m1
TBP	Hs00920495_m1

Supplementary Table 3

Functional classification of genes deregulated in stem cells depleted in COSMOC expression

Cluster 1 (Enrichment Score: 3.21)					
Term	%	p-Value	Genes	Fold Enrichment	Benjamini
GO:0006695~cholesterol biosynthetic process	1,72E+00	1,01E-05	EBP, SQLE, DHCR7, INSIG1, FDPS, LSS, IDI1, NSDHL, DHCR24	8,21	6,51E-03
GO:0033489~cholesterol biosynthetic process via desmosterol	5,75E-01	0,0047697	EBP, DHCR7, DHCR24	26,01	4,44E-01
GO:0033490~cholesterol biosynthetic process via lathosterol	5,75E-01	0,0047697	EBP, DHCR7, DHCR24	26,01	4,44E-01
Cluster 1 (Enrichment Score: 2,93)					
Term	%	p-Value	Genes	Fold Enrichment	Benjamini
GO:0006334~nucleosome assembly	3,07E+00	1,64E-06	H1F0, HIST1H4L, HIST1H1C, HIST1H1B, NAP1L5, TSPYL4, TSPYL5, HIST1H2BM, HIST1H3B, HIST1H3C, H2AFY, HIST1H4C, HIST1H4D, HIST1H3F, ASF1B, HIST1H4H	4,66	2,11E-03
GO:0006335~DNA replication-dependent nucleosome assembly	1,72E+00	2,54E-06	HIST1H4L, RBBP4, HIST1H3B, HIST1H3C, HIST1H4C, HIST1H4D, ASF1B, HIST1H3F, HIST1H4H	9,75	2,18E-03
GO:0045814~negative regulation of gene expression, epigenetic	1,92E+00	1,10E-05	HIST1H4L, RBBP4, JARID2, HIST1H3B, H2AFY, HIST1H3C, HIST1H4C, HIST1H4D, HIST1H3F, HIST1H4H	6,94	5,65E-03
GO:0032200~telomere organization	1,34E+00	9,73E-05	HIST1H4L, HIST1H3B, HIST1H3C, HIST1H4C, HIST1H4D, HIST1H3F, HIST1H4H	8,99	3,52E-02
GO:0051290~protein heterotetramerization	1,53E+00	2,05E-04	HIST1H4L, RRM2, HIST1H3B, HIST1H3C, HIST1H4C, HIST1H4D, HIST1H3F, HIST1H4H	6,45	6,40E-02
GO:0045815~positive regulation of gene expression, epigenetic	1,72E+00	3,83E-04	HIST1H4L, HIST1H3B, H2AFY, HIST1H3C, DDX21, HIST1H4C, HIST1H4D, HIST1H3F, HIST1H4H	5,03	1,04E-01
GO:0006352~DNA-templated transcription, initiation	1,34E+00	4,39E-04	TAF13, HIST1H4L, TAF12, HIST1H4C, HIST1H4D, TCF4, HIST1H4H	6,94	1,07E-01
GO:0000183~chromatin silencing at rDNA	1,34E+00	5,99E-04	HIST1H4L, HIST1H3B, HIST1H3C, HIST1H4C, HIST1H4D, HIST1H3F, HIST1H4H	6,56	1,31E-01
GO:0006336~DNA replication-independent nucleosome assembly	1,15E+00	7,70E-04	HIST1H4L, RBBP4, HIST1H4C, HIST1H4D, ASF1B, HIST1H4H	8,00	1,52E-01
GO:0045653~negative regulation of megakaryocyte differentiation	7,66E-01	0,01400183	HIST1H4L, HIST1H4C, HIST1H4D, HIST1H4H	7,71	6,25E-01
GO:0031047~gene silencing by RNA	1,72E+00	0,01482966	POLR2G, HIST1H4L, RAN, HIST1H3B, HIST1H3C, HIST1H4C, HIST1H4D, HIST1H3F, HIST1H4H	2,81	6,37E-01
GO:0044267~cellular protein metabolic process	1,72E+00	0,02069608	HIST1H4L, GSN, HIST1H3B, HIST1H3C, HIST1H4C, HIST1H4D, HIST1H3F, CCDC59, HIST1H4H	2,65	6,98E-01
GO:0016233~telomere capping	7,66E-01	0,02734151	HIST1H4L, HIST1H4C, HIST1H4D, HIST1H4H	6,03	7,34E-01
GO:0034080~CENP-A containing nucleosome assembly	9,58E-01	0,03453138	HIST1H4L, RBBP4, HIST1H4C, HIST1H4D, HIST1H4H	4,03	7,73E-01
GO:1904837~beta-catenin-TCF complex assembly	7,66E-01	0,12594284	HIST1H4L, HIST1H4C, HIST1H4D, HIST1H4H	3,23	9,43E-01
GO:0006303~double-strand break repair via nonhomologous end joining	7,66E-01	0,26407247	HIST1H4L, HIST1H4C, HIST1H4D, HIST1H4H	2,24	9,90E-01
GO:0098609~cell-cell adhesion	4,80E+00	1,20E-06	HDLBP, LIMA1, STK38, CNN3, DIAPH3, WASF2, EFHD2, PICALM, FAM129B, EHD1, TES, RAN, SWAP70, CBL, EIF2S3, TRIM25, ELMO2, KRT18, KIAA1524, HIST1H3B, HIST1H3C, TMPO, HIST1H3F, YKT6, ADD1	3,20	3,10E-03
GO:0007568~aging	3,07E+00	8,52E-04	LITAF, KL, FADS1, TGFB3, CNP, DCN, CCL5, PPARGC1A, GCLM, VCAM1, EDNRA, TFRC, CTGF, GSN, AMFR, CASP2	2,72	1,55E-01
GO:0055114~oxidation-reduction process	5,94E+00	1,91E-03	CYP2U1, CYB5R1, UGDH, AKR1C3, VCAM1, PTGIS, DHCR7, PLOD3, DHCR24, NSDHL, ACADM, PCBD1, FADS1, SCD, QDPR, FADS2, CYB5A, CYP2E1, DHRS1, ADI1, OGFOD1, RDH10, DHFR, NNT, SQLE, RRM2, CYBRD1, TXNRD1, KIAA1191, ALDH9A1, RETSAT	1,82	2,80E-01

References

1. Feron, F., Gepner, B., Lacassagne E., Stephan, D., Mesnage, B., Blanchard, M. P. et al., Olfactory stem cells reveal MOCOS as a new player in autism spectrum disorders. Mol Psychiatry. 2016;21:1215-1224.
2. Li, W., Sun, W., Zhang, Y., Wei, W., Ambasudhan, R., Xia, P. et al., Rapid induction and long-term self-renewal of primitive neural precursors from human embryonic stem cells by small molecule inhibitors. Proc Natl Acad Sci USA. 2011;108:8299-8304.
3. Sancho-Martinez, I., Nivet, E., Xia, Y., Hishida, T., Aguirre, A., Ocampo, A. et al., Establishment of human iPSC-based models for the study and targeting of glioma initiating cells. Nat Commun. 2016;7:10743.
4. Bardy, C., van den Hurk, M., Eames, T., Marchand, C., Hernandez, R. V., Kellogg, M. et al., Neuronal medium that supports basic synaptic functions and activity of human neurons in vitro. Proc Natl Acad Sci USA. 2015; 112:E2725-2734.
5. Chiocchetti, A. G., Haslinger, D., Stein, J. L., de la Torre-Ubieta, L., Cocchi, E., Rothamel, T. et al., Transcriptomic signatures of neuronal differentiation and their association with risk genes for autism spectrum and related neuropsychiatric disorders. Transl Psychiatry. 2016;6:e864.
6. Ran F. A., Hsu, P. D., Wright, J., Agarwala, V., Scott, D. A. & Zhang, F. Genome engineering using the CRISPR-Cas9 system. Nat Protoc. 2013;8:2281-2308.
7. Jeong, J. Y. Yim, H. S., Ryu, J. Y., Lee, H. S., Lee, J. H., Seen, D. S. et al., One-step sequence- and ligation-independent cloning as a rapid and versatile cloning method for functional genomics studies. Appl Environ Microbiol. 2012;78:5440-5443.
